# Flyway connectivity and exchange primarily driven by moult migration in geese

**DOI:** 10.1186/s40462-019-0148-6

**Published:** 2019-01-31

**Authors:** A. Kölzsch, G. J. D. M. Müskens, P. Szinai, S. Moonen, P. Glazov, H. Kruckenberg, M. Wikelski, B. A. Nolet

**Affiliations:** 1 Department of Migration and Immuno-Ecology, Max Planck Institute for Ornithology, Am Obstberg 1, 78315 Radolfzell, Germany; 2 Department of Biology, University of Konstanz, Universitätsstraße 10, 78464 Konstanz, Germany; 3 Institute for Wetlands and Waterbird Research e.V, Am Steigbügel 13, 27283 Verden (Aller), Germany; 4 Team Animal Ecology, Wageningen Environmental Research, Wageningen University & Research, Droevendaalsesteeg 3-3A, 6708 PB Wageningen, The Netherlands; 5 Balaton-felvidéki National Park Directorate, Kossuth utca 16, Csopak, 8229 Hungary; 6 Bird Ringing and Migration Study Group of BirdLife Hungary, Koltő utca 21, Budapest, 1121 Hungary; 7 Institute of Avian Research, An der Vogelwarte 21, 26386 Wilhelmshaven, Germany; 8 Institute of Geography, Russian Academy of Sciences, Staromonetnyi per. 29, 119017 Moscow, Russia; 9 Department of Animal Ecology, Netherlands Institute of Ecology (NIOO-KNAW), Droevendaalsesteeg 10, 6708 PB Wageningen, The Netherlands; 10 Department of Theoretical and Computational Ecology, Institute for Biodiversity and Ecosystem Dynamics, University of Amsterdam, Science Park 904, 1098 XH Amsterdam, The Netherlands

**Keywords:** Dynamic Brownian bridges, GPS tracking, Greater white-fronted goose, Long-distance moult migration, Migratory connectivity, Population exchange, Population overlap, Taimyr peninsula

## Abstract

**Background:**

For the conservation and management of migratory species that strongly decrease or increase due to anthropological impacts, a clear delineation of populations and quantification of possible mixing (migratory connectivity) is crucial. Usually, population exchange in migratory species is only studied in breeding or wintering sites, but we considered the whole annual cycle in order to determine important stages and sites for population mixing in an Arctic migrant.

**Methods:**

We used 91 high resolution GPS tracks of Western Palearctic greater white-fronted geese (*Anser A. albifrons*) from the North Sea and Pannonic populations to extract details of where and when populations overlapped and exchange was possible. Overlap areas were calculated as dynamic Brownian bridges of stopover, nest and moulting sites.

**Results:**

Utilisation areas of the two populations overlapped only somewhat during spring and autumn migration stopovers, but much during moult. During this stage, non-breeders and failed breeders of the North Sea population intermixed with geese from the Pannonic population in the Pyasina delta on Taimyr peninsula. The timing of use of overlap areas was highly consistent between populations, making exchange possible. Two of our tracked geese switched from the North Sea population flyway to the Pannonic flyway during moult on Taimyr peninsula or early during the subsequent autumn migration. Because we could follow one of them during the next year, where it stayed in the Pannonic flyway, we suggest that the exchange was long-term or permanent.

**Conclusions:**

We have identified long-distance moult migration of failed or non-breeders as a key phenomenon creating overlap between two flyway populations of geese. This supports the notion of previously suggested population exchange and migratory connectivity, but outside of classically suggested wintering or breeding sites. Our results call for consideration of moult migration and population exchange in conservation and management of our greater white-fronted geese as well as other waterfowl populations.

## Background

Humans are exerting an ever increasing impact on the environment, and migratory animal populations are declining worldwide as a result [1]. Migrants depend on more than one area, making them vulnerable to changes in any of these areas, as well as to changes in the connections between these areas. An understanding of the population consequences of these changes requires a proper delineation of populations, and hence a notion about whether or not mixing occurs between animals from certain breeding and non-breeding areas, a concept know as migratory connectivity [2]. This concept quantifies the extent to which individuals from one breeding population migrate to the same non-breeding locations or vice versa [2, 3].

However, the world is not changing for the worse for all migratory species. Many goose populations are thriving because of environmental changes, in particular in their wintering and stopover areas, that enhance their survival [4–6] and, through carry-over effects, reproduction [7, 8]. In some cases, goose populations are growing so much that management actions are implemented or proposed [9, 10]. In migratory geese, management actions taken at one place and time may affect the presence and behaviour of birds at other places and times along the migratory route [11, 12]. A delineation of the flyway population, including an estimate of the exchange of individuals with other flyway populations, should therefore become an integral part of conservation and control processes. This is even more important if population dynamics and demography are unclear and seemingly stable populations might be cryptic sinks [13] and lead to population shifts [14].

The origin of migratory waterfowl and the exchange between flyways has previously been documented based on ring re-encounters [14–17], isotopic signatures [18, 19] or genetic data [20–22]. Ringing and isotopic data can shed light on short-term exchanges, whereas genetic data usually only detect more permanent immigrations that led to gene flow. Lately, genetic samples from large numbers of sites throughout the annual cycle (genoscapes) allow also the quantification of more recent dispersal, but depend on large data sets and previous knowledge of the location of key sites [23]. Although these are all useful methods to determine the extent of exchange between flyways, they at most provide coarse information about when and where such exchange happens.

By equipping birds with tracking devices, more and more data are being gathered on migration routes [24]. Such data have recently also been used to study migratory connectivity [15, 25, 26]. When birds from adjacent flyways are followed, their tracks might indicate areas of flyway overlap, which may be the potential areas where exchanges take place. As tracking devices are costly, sample size often prevents the observation of actual exchange events, but, at the positive side, such tracking is much less prone to bias than ringing data, for which reporting rates may vary spatially [27].

For the greater white-fronted geese (*Anser A. albifrons*) in the Western Palearctic, different flyway populations have traditionally been distinguished, based on their spatially segregated wintering quarters [28]. The two most westerly ones are the North Sea population, with its main wintering area in the Netherlands and Germany, and the Pannonic population, with its main wintering area in Hungary [29]. After a steady increase since the 1960’s, numbers of the North Sea population have stabilised at 1.2 million birds since 2001 [30]. However, breeding success has been low since 1991 and seems to be further decreasing to about 10–25%, presumably due to changes in the breeding area [28, 30]. Pannonic population numbers are significantly lower, but are based on rather incomplete counts. They show a continuous population growth from 1986 until 2012 with an average of 140,000 birds in the later years. Breeding success seems to be somewhat higher than for the North Sea population, but not significantly so [30].

Some twenty years ago, it has been suggested that the two populations mix to such a degree that increases in numbers of one population were linked to decreases in numbers in the other population [16]. A recent analysis of ringing data indeed found high rates of exchange (12–23%), occurring between winter seasons rather than within winter seasons, but the analysis was strongly hampered by the enormous difference in re-sighting and reporting probabilities between the main wintering quarters [30].

We tracked greater white-fronted geese from both the North Sea and Pannonic flyway population to determine flyway overlap, and in the expectation to see at least some overlap, where and when exchange (potentially) occurs outside the winter season. Although our results indicated a slight overlap in migration routes to and from the breeding grounds, they emphasize the importance of the moult migration of failed breeders as a mechanism of flyway exchange.

## Methods

### GPS tracking

Greater white-fronted geese were caught during moult on Kolguev Island in Russia (August 2013 and 2016) or in winter in The Netherlands and northern Germany (November–February 2013/14–2016/17) as well as in Hungary (November–February 2012/13, 2015/16 and 2016/17). During moult, standing nets were used, whereas cannon netting with artificial decoys or clap nets with live decoys were applied during winter. We equipped a subset of the caught geese, all adults, with solar GPS (global positioning system) tags of various designs and manufacturers (Table 1), either attached as backpack with Teflon harness [31] or as integrated neckband. Duty cycles were set to 1 GPS position per hour (Microwave tags) or per 15 min (all other tags), but were decreased down to 1 position per 1, 4 or 12 h, respectively, in times of low energy levels due to reduced sunlight. As white-fronted geese stay year-round in pairs or families, no sex effects were expected on timing and space use. In order to ensure independence, all tracks were single years from individuals that did not migrate together. In total, we could use data of 6 males and 4 females tracked in the Pannonic population (i.e., from Hungary), and 51 males and 30 females in the North Sea population (i.e., from The Netherlands, northern Germany or Kolguev Island).

**Table 1:** Tagging details and selected successful tracks of greater white-fronted geese

Catch location	Year of catch^b^	Tag type	Tag weight	Tag manufacturer	Tag attachment	Number of successful tracks	Years of selected tracks^c^
Kolguev Island	2013	solar GSM/GPS	45 g	E-obs GmbH	backpack with Teflon harness	4 ♀, 6 ♂	9 × 2014, 1 × 2015
	2013	solar UHF/GPS	35 g	Univ. of Konstanz	integrated neckband	1 ♀, 12 ♂	9 × 2014, 4 × 2015
	2016	solar GPRS/GPS	38 g	Madebytheo	integrated neckband	7 ♀, 5 ♂	12 × 2017
Netherlands/ Northern Germany^a^	2013/14 and 2014/15	solar GSM/GPS	45 g	E-obs GmbH	backpack with Teflon harness	3 ♀, 4 ♂	3 × 2014, 4 × 2015
	2015/16 and 2016/17	solar GPRS/GPS	38 g	Madebytheo	integrated neckband	15 ♀, 24 ♂	3 × 2015, 21 × 2016, 15 × 2017
Hungary	2012/13	solar Argos/GPS	45 g	Microwave Telemetry Inc.	backpack with Teflon harness	5 ♂	5 × 2013
	2015/16 and 2016/17	solar GPRS/GPS	38 g	Madebytheo	integrated neckband	4 ♀, 1 ♂	1 × 2016, 4 × 2017

^a^ in first two seasons in The Netherlands only

^b^ summer or winter season

^c^ selection criteria: no gaps > 48 h, 1 March – 15 November

### Extraction of migration stopovers, nest and moult sites

For detection of overlap of the two (in winter disparate) populations, we selected tracks between 1 March – 15 November, thus including spring migration, breeding, moult and autumn migration. We extracted migration stopovers as sites where geese spent at least 2 days in an area of 30 km radius [32] during spring (1 March – 1 June) and autumn (15 August – 15 November). Nesting sites were defined where geese spent at least 10 days in an area of 2 km [33] during 15 May – 15 July, thus including successful and failed breeding events. Failed breeders were determined as birds moving > 100 km from their nesting sites in the time of 10–26 days after nest initiation. Moulting sites were determined where geese spent at least 21 days in an area of 30 km radius between 1 July and 1 September.

### Detection of population overlaps

For each of those periods and for each individual goose, we calculated the utility distribution area with dynamic Brownian bridges (function “brownian.bridge.dyn” in R-package “move” [34], window size = 31, margin size = 11) as raster maps with grid cell size 5 × 5 km for migration stopovers and 750 × 750 m for nesting and moulting sites. In order to combine those to population level utility distributions, we defined “used grid cells” as those where at least one individual bird was expected to be for > 15 min (threshold based on data resolution). For each population and the four periods (see *Extraction of migration stopovers, nest and moult sites*) we determined overlapping used grid cells and calculated the proportion of cells that overlapped. For further analyses, the overlapping sites were grouped by period and distance into regions. In order to detect if the region use by members of the two populations also overlapped in time, for each such region we extracted the number of individuals per population that used it (i.e. had GPS positions less than 30 km from the cells’ borders; threshold based on stopover site definition) during or outside the stopover/nesting/moulting time and quantified the time and duration of use and overlap.

### Example tracks for population switching

The data set includes tracks of two adult males that were caught in either The Netherlands (2014/15; goose NK3) or northern Germany (2015/16, goose 424), and were observed to switch to wintering in Hungary in one or more subsequent years. We present their tracks, migration stopovers, nesting and moulting sites (as above) during the two available years of data each and indicate likely regions of population switching.

## Results

### Migratory stopovers, nesting and moulting sites

**Fig. 1:**
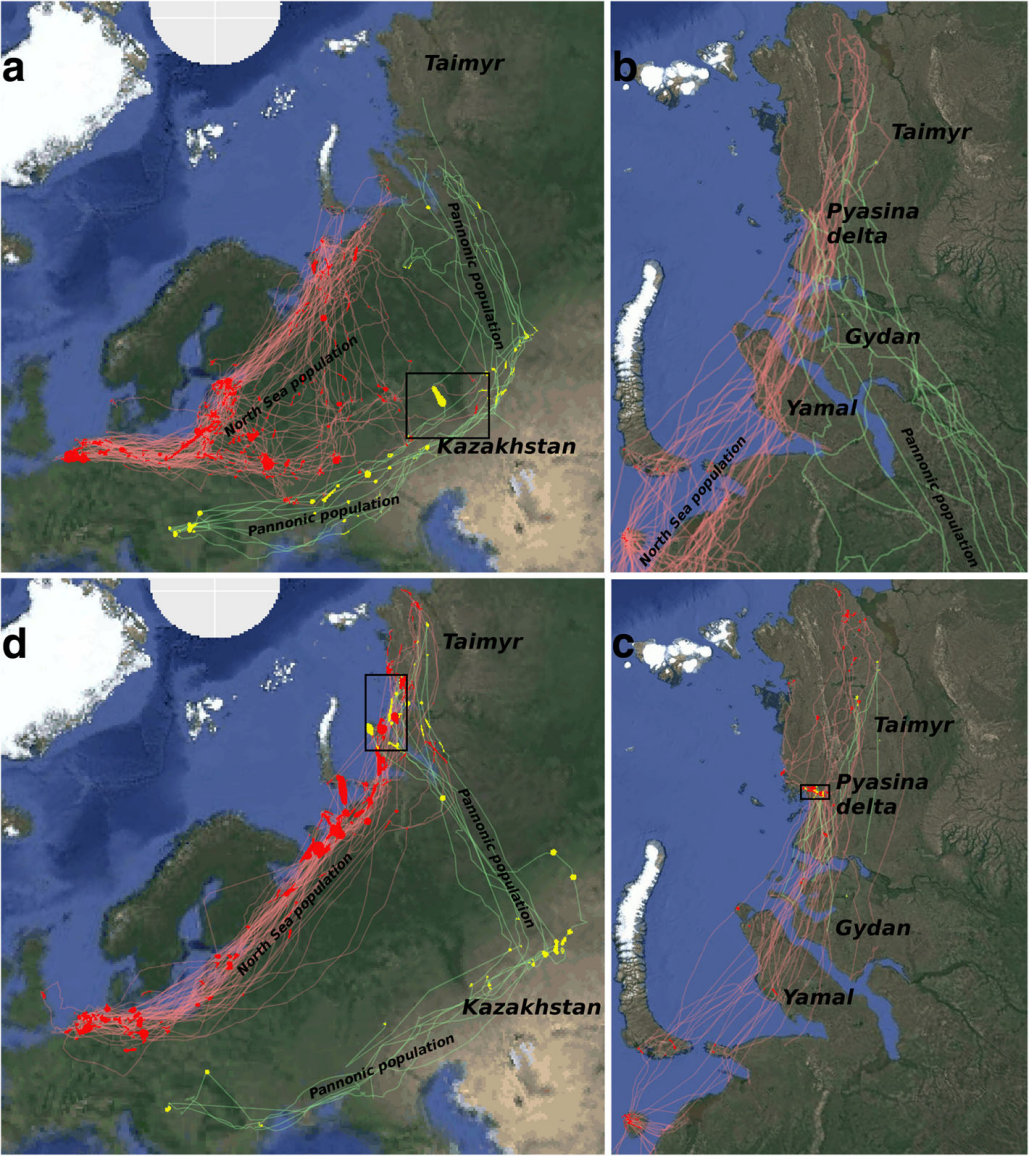
GPS movement tracks of geese from the North Sea population (red lines) and Pannonic population (green lines) with dynamic Brownian bridge utilisation distribution cells of stopover, nest and moult sites (red cells for North Sea population, yellow cells for Pannonic population). Clockwise: (**a**) Spring migration tracks and stopovers, (**b**) nesting sites with tracks of 15 May – 15 July, (**c**) moulting sites with tracks of 1 July – 1 September and (**d**) autumn migration tracks and stopovers. Outlines are of overlap regions (see Fig. 2). Note that there is no overlap of nesting sites (North Sea population: Kolguev – Yamal; Pannonic population: Gydan and Taimyr). Tracks during moult (**c**) are not clearly separated by population

Geese of the North Sea population used on average 6.0 (± 0.2, standard error, range: 1–11) stopover sites during spring migration and 4.9 (± 0.2, range: 1–8) stopover sites during autumn migration. The stopovers were rather evenly distributed along the flyway, especially in spring (Fig. 1a, d). Geese of the Pannonic population used 7.0 (± 0.5, range: 5–9) stopover sites during spring and 5.7 (± 0.7) stopover sites during autumn migration. Stopovers of Pannonic geese were more concentrated in Kazakhstan and not as evenly distributed over the flyway (Fig. 1a, d).

From the North Sea population we detected a nesting site for 54 birds (67%) and a moulting site for 57 birds (70%). Of the 54 breeders, 20 birds (37%) failed and moved up to 2000 km to moult on Taimyr peninsula (Fig. 1b, c). Additionally, of the 27 non-breeders, 9 birds (33%) moulted on Taimyr, so in total 36% of the birds tracked in the North Sea population moulted there. From the Pannonic population we extracted a nesting site for 5 birds (50%) and a moulting site for 9 birds (90%). Two birds nested on Gydan peninsula and 3 birds nested on Taimyr. All 9 (i.e. 100%) extracted moulting sites were situated on Taimyr (from the two birds nesting on Gydan, one likely failed breeding and moved to Taimyr, whereas from the other bird a moulting site was missing).

The total area (cumulated from the dynamic Brownian bridge utilisation distribution cells; Fig. 1) that geese from the North Sea population used during migration was large and very similar between spring and autumn (Table 2). This is notable, because geese from this population migrate in a much wider front in spring than in autumn (Fig. 1). For the Pannonic population this total area was also similar between spring and autumn, but much smaller than for the North Sea population. However, because average total used area of individual birds was consistently larger for the Pannonic population (in spring Pannonic: 220 vs. North Sea: 127 and autumn Pannonic: 243 vs. North Sea: 151), we conclude that this is due to smaller sample size. In comparison to total migration stopover area, moulting site total area was much smaller for both populations (Table 2).

**Table 2:** Details of dynamic Brownian bridge utilisation distribution cells and population overlap

	North Sea population		Pannonic population	
	Total area^a^	Overlap proportion^b^	Total area^a^	Overlap proportion^b^
Spring migration stopovers	222,125 km^2^	0.05%	55,025 km^2^	0.18%
Moult sites	1741 km^2^	1.52%	56,050 km^2^	16.15%
Autumn migration stopovers	255,425 km^2^	0.84%	164 km^2^	3.84%

^a^ total area of dynamic Brownian bridge utilisation distributions cells

^b^ proportion of cells of each population that overlapped with the other population during spring migration, moult or autumn migration

### Regions and timing of population overlap

**Fig. 2:**
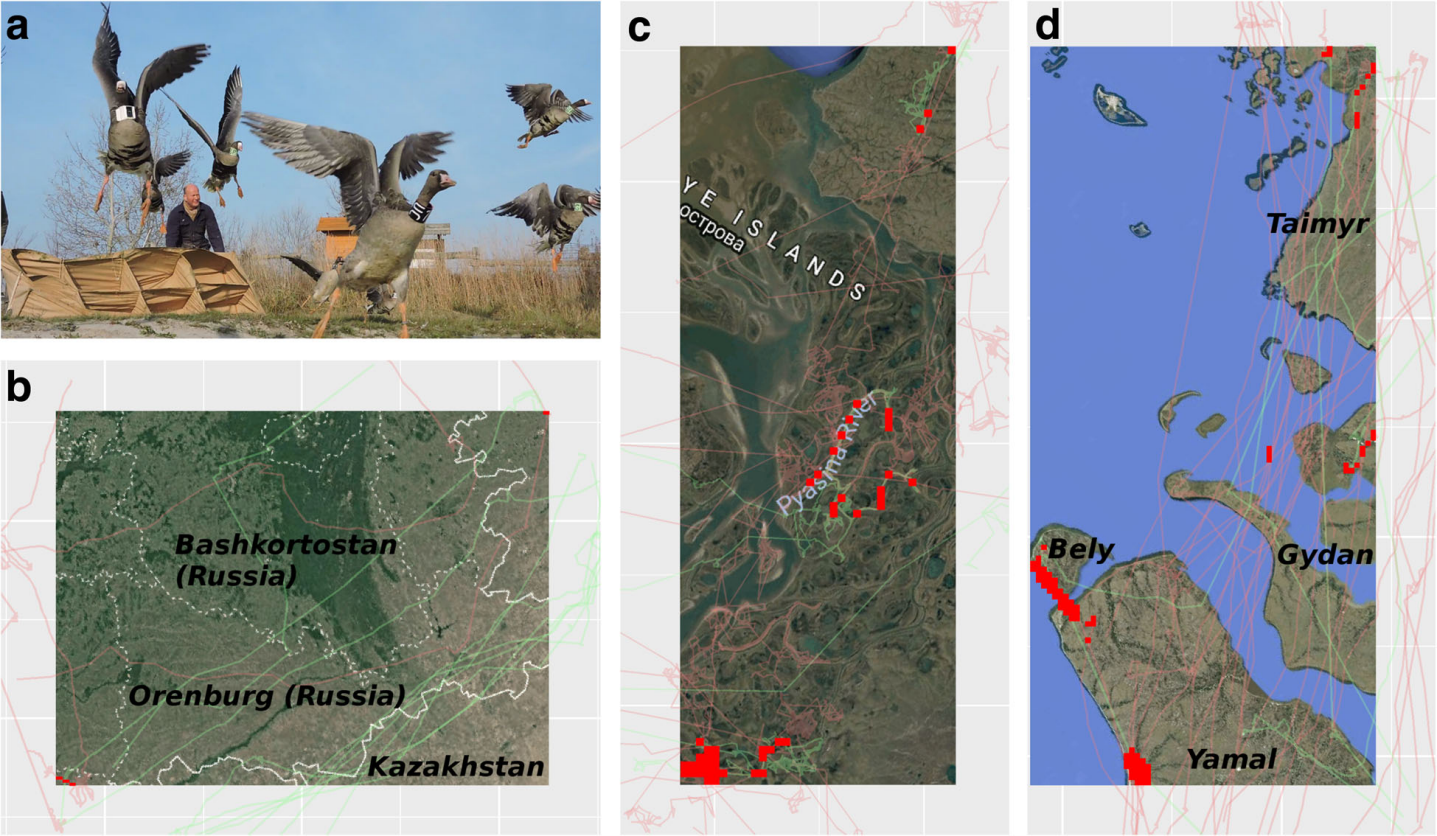
**a** Ringed and tagged greater white-fronted geese being released in Hungary 2016/17. Overlap regions (red cells) and GPS tracks (red for North Sea population, green for Pannonic population) towards and from those regions during (**b**) spring migration (North-Western Kazakhstan), (**c**) moult (Pyasina delta) and (**d**) autumn migration (Pyasina delta, Gydan peninsular, Bely island and Yamal). See outlines of those regions indicated in Fig. 1 **a, c, d**. Note that overlap regions in (**b**) are in the top-right and bottom-left corners of the map. GPS tracks are not clearly separated by population at this scale

**Fig. 3:**
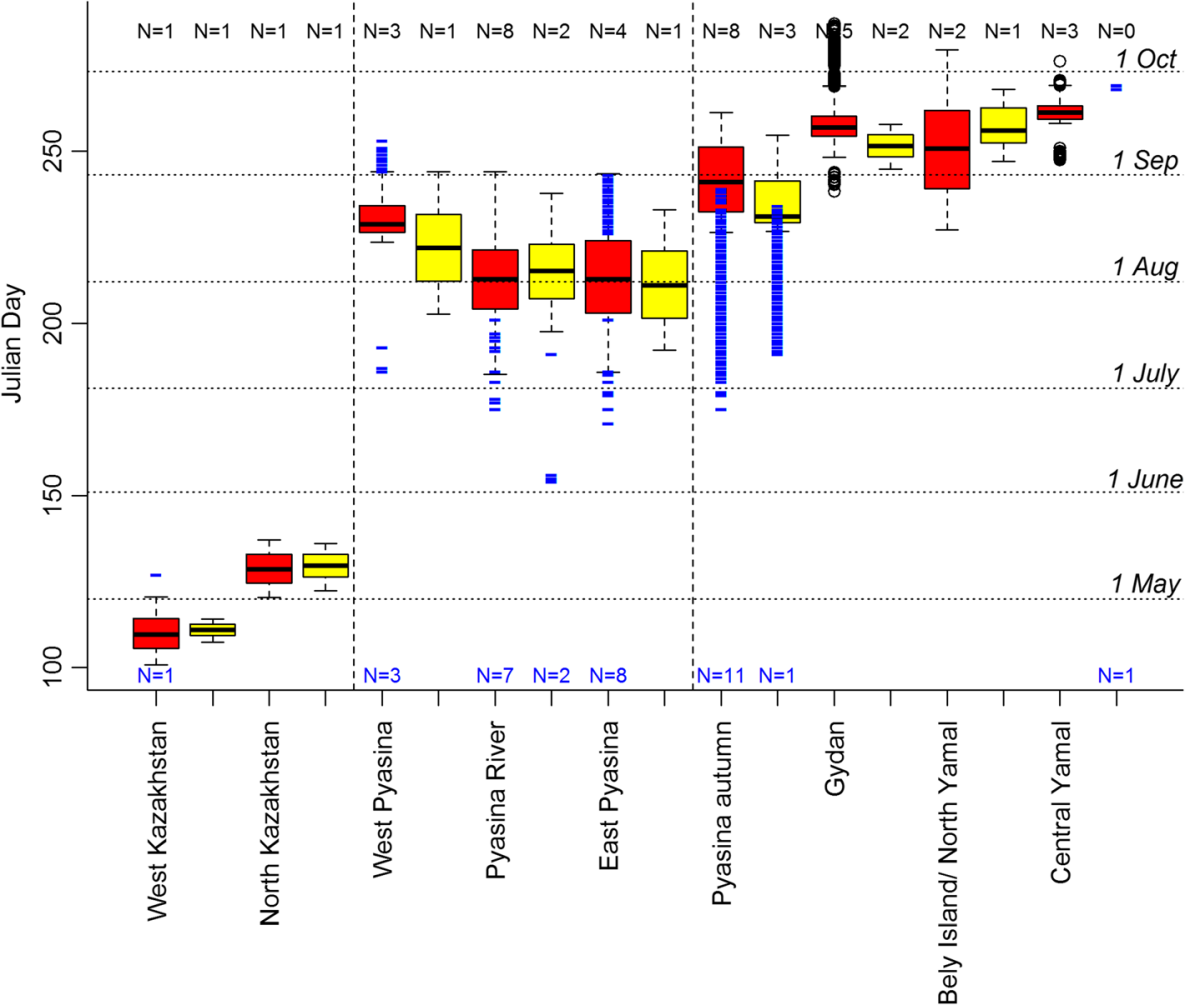
Boxplot of times (in Julian Days, see 1st of each month indicated on the right) that tracked geese were stopping in each of the named overlap regions (red for North Sea population, yellow for Pannonic population). Vertical dashed lines separate spring migration, moult and autumn migration sites. Sample sizes (number of individuals) are indicated above. Small blue lines indicate days when geese were passing through the respective overlap region without stopping or moulting there, sample sizes are indicated below in blue

The two populations of geese had overlapping sites during spring migration stopover, moult and autumn migration stopover, but not for nesting sites. The two overlap regions during spring migration were very small and both located close to the border between Russia and Kazakhstan (Fig. 2b), in the following named West Kazakhstan and North Kazakhstan. Those overlap regions summed to less than 1% of the total spring migration stopover area of both populations (Table 2). From each population, only one bird stopped in either of the two regions, but timing overlapped well (Fig. 3). Geese stayed for 7–20 days (Table 3), so there might have been possibility for exchange.

**Table 3:** Durations (in days) spent in overlap regions by geese of each population

	North Sea population	Pannonic population
West Kazakhstan	19.8	6.8
North Kazakhstan	16.6	13.7
West Pyasina	21.8 (±12.7, N = 3)	41.3
Pyasina River	35.8 (±2.0, N = 8)	34.9 (±1.6, N = 2)
East Pyasina	33.6 (±9.1, N = 4)	40.8
Pyasina autumn	15.7 (±4.3, N = 8)	13.8 (±7.1, N = 3)
Gydan	17.3 (±4.7, N = 5)	6.5 (±6.5, N = 2)
Bely Island/ North Yamal	50.8 (±1.4, N = 2)	21.0
Central Yamal	5.3 (±3.7, N = 3)	–

Standard error and sample sizes (number of individuals in the region) indicated in brackets, if none are indicated sample size is N = 1 and therefore no standard error

All overlapping sites during moult were situated on Taimyr at the Pyasina delta (Fig. 2c), for comparison we have split them into three regions: West Pyasina, Pyasina River and East Pyasina. The proportion of those overlap cells from the Pannonic population was comparatively high (16.2%; Table 2) and also from the North Sea population it was higher than for the migration stopovers (1.5%). This indicates a small area for a high density of North Sea and Pannonic birds that moult in the Pyasina delta and much possibility for exchange. Timing overlapped well for the many tracked birds that moulted there (Fig. 3) and staying duration was 22–41 days (Table 3). Several birds, especially of the North Sea population, shortly passed by before or after moulting there or elsewhere.

There were large areas of overlapping autumn migration stopovers close to the breeding and moulting sites, some still in the Pyasina delta, others on the adjacent peninsulas and islands. We define four regions for further use: the Pyasina delta (hereafter called Pyasina autumn), Gydan, Bely Island/North Yamal and Central Yamal (Fig. 2d). The proportion of overlap cells were again small, but larger than for spring migration stopovers (Table 2), which might be due to the closeness to the moulting sites. The timing of region use overlapped well for the two populations (Fig. 3), maybe less so for Central Yamal due to low sample size. Staying durations were more variable than during spring or moult, namely 5–51 days (Table 3). Many geese seemed to pass by the Pyasina autumn region long before autumn migration, but these were birds that had moulted in the same area before, now likely fuelling for autumn migration flight (Fig. 3).

### Observed population switches

**Fig. 4:**
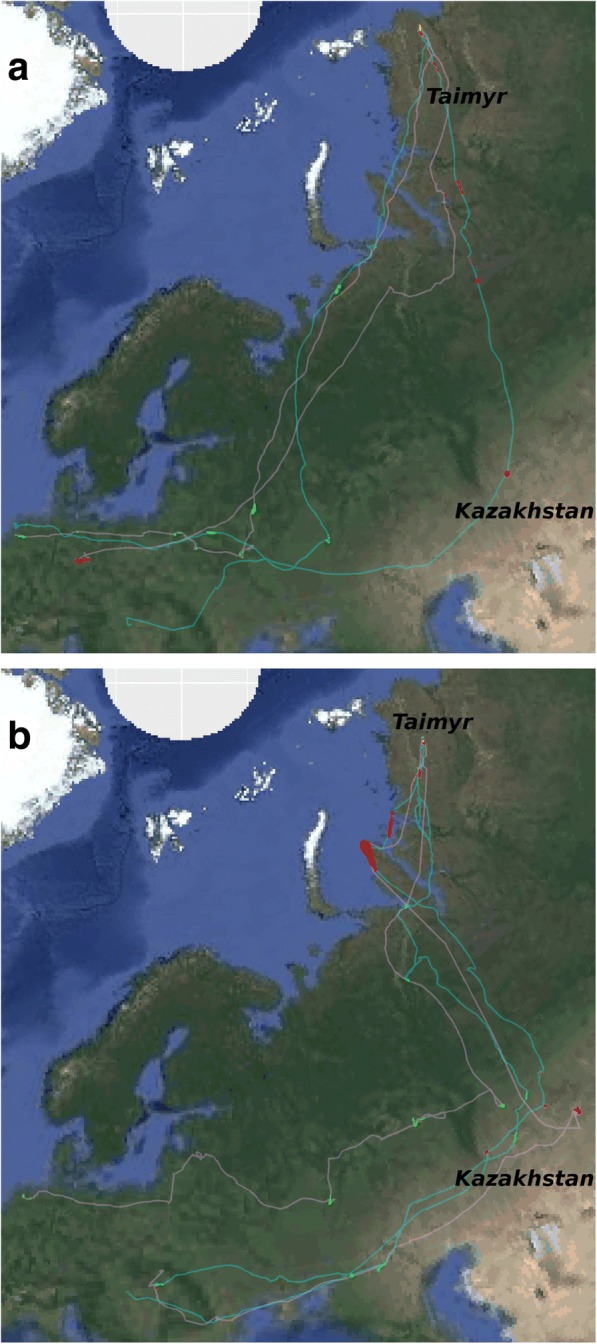
GPS tracks of two adult male geese that switched from the North Sea population to the Pannonic population. Green marks stopover utilisation cells during spring migration, yellow marks cells during moult and red cells during autumn migration. **a** Tracks of goose NK3 are shown for 2015 (grey) and 2016 (cyan). It likely switched during moult in 2016. **b** Tracks of goose 424 are shown for 2016 (grey) and 2017 (cyan). It likely switched during spring migration or moult in 2016

The first goose (NK3) for which we observed a population switch, followed a southern route after departing from The Netherlands during its first tracked spring migration (2015). We could not detect a nesting attempt, but instead it moved far to Eastern Taimyr for moult (Fig. 4a). The subsequent autumn migration started off far south, close to the tracks of the Pannonic population. However, that year it changed direction, crossed the Ural mountains westward and joined the North Sea population birds towards its previous wintering site in The Netherlands. The second tracked spring migration (2016) was even further south, but still in the flyway of the North Sea population, again skipping to nest and towards Eastern Taimyr for moult. Also that year’s autumn migration started off towards the south, however this time the bird stayed in the flyway of the Pannonic population east of the Ural mountains, doing a stopover in Northern Kazakhstan (see other Pannonic birds in Fig. 1c), ending up in Hungary to winter. It is likely that this bird switched from the North Sea population to the Pannonic population during moult in 2016. Unfortunately, we could not follow its spring migration in 2017 after it was shot in Hungary in January 2017.

The second goose (424) showed an even clearer population switch in its first tracked year (2016). It migrated from its wintering site in The Netherlands on a southern spring route via the Ukraine and Kazakhstan (Fig. 4b), already close to the Pannonic population’s flyway, which overlaps with the North Sea population’s flyway there. However, when approaching the Arctic coast it crossed the Ural mountains westward like some other North Sea birds (Fig. 1a) approaching its moulting site on Central Taymir via the North Sea flyway, along the Arctic coast, Yamal and Gydan peninsula. We could not detect a nesting attempt that year nor the following. Goose 424 started its first tracked autumn migration along the Arctic coast, stopping long on Bely Island and Northern Yamal. Then it turned south and joined the flyway of the Pannonic population, staying east of the Ural mountains and moving via Kazakhstan to a wintering site in Hungary. So it has switched from the North Sea population to the Pannonic population in 2016 during moult or autumn migration. During its next year’s spring and autumn migration it strictly followed the migration routes of the Pannonic population, indicating that the switch was long-term.

## Discussion

Using GPS tracking data of greater white-fronted geese of two flyway populations we have shown that there are areas where birds from both populations overlap in time and space. Few such overlaps occurred during spring and autumn migration, but the most important one was during moult in the Pyasina delta on Taymir peninsula. There, most non-breeders and failed breeders of the North Sea population met with geese, breeders and non-breeders alike, from the Pannonic population, leading to possible exchange events. We were able to track two cases of individual switching, likely at moulting sites or at least following migration from these sites. Further, we could show that those birds completely used the new flyway, one bird even for two seasons, suggesting long-term or permanent population exchange.

### The importance of moult migration

Previous research documented population exchanges in swans and geese during migration [15] or in winter [14], but in our case moult migration of failed or non-breeders was the key phenomenon creating overlap between the two flyways. Such extensive moult migrations are a well-known phenomenon in waterfowl [35–40], but have not been recognized before as a key factor in flyway population exchange. In support of our findings, recently a mixed moult site of two Northern American populations of greater white-fronted geese has been detected by ringing data [41].

During wing moult, waterfowl shed all their flight feathers simultaneously, and cannot fly for some weeks until the flight feathers have regrown. Waterfowl are precocial and nidifugous species, meaning that their young are mobile and leave the nest shortly after hatch, weeks before they can fly. During this period, the young are accompanied by their parents, which also cannot fly at that time as they undergo wing moult soon after breeding. So, only birds that refrain from breeding or that fail their breeding attempt are free to move to moulting areas at flying distance. In our study, moult migrations were up to 2000 km long. It is generally thought that such moult migrations take the birds to areas that are relatively safe [37] and/or offer good feeding opportunities that become available later in the season than the breeding areas [36].

### Overlap in space and time

In the case of greater white-fronted geese, overlap outside the moulting phase was rather limited. In fact, the flyways ran much less close to each other than previously thought [29]. The birds from the North Sea and Pannonic population basically flew west and east from the Ural mountains, and this mountain range therefore forms a natural barrier between the two flyways. Both the wintering and breeding ranges are also separated in space. Wintering areas were located in two lowlands separated by mountainous regions including the Alps, and nesting areas were west and east of the foothills of the Ural mountains. However, non-breeders, or birds with failed breeding events, in particular from the North Sea population, fly further to the east, north of the Ural foothills, to moult at Taimyr peninsula, where geese from the Pannonic population breed and moult. As a result, the largest overlap between the two flyways occurs at this peninsula.

Timing is crucial when considering migratory connectivity [42]. In North-America, within a single flyway, three populations of greater white-fronted geese largely overlap in space, but apart from using different breeding areas, differ largely in timing of stopover as well as habitat use within stopovers [43, 44]. On the contrary, greater white-fronted geese from the two populations in Europe followed different routes, but did not differ in timing where they met. However, the birds from the North Sea population gradually follow the advancement of spring on their way to the breeding grounds [32], while those from the Pannonic population stage a long time in Kazakhstan before jumping towards their breeding grounds in the tundra. This jumping over the taiga zone has also been described for swans and geese further to the east in Asia [45, 46], and is probably due to the lack of suitable stopover sites in this vast region.

### Flyway switching in social migrants

To some extent the exchange between flyway populations is surprising, as geese are social migrants like swans and cranes, species in which the migration route is learned from more experienced individuals, often the parents [47, 48]. Especially greater white-fronted geese are known for their very long family bonds that can last well beyond the return migration [49–51]. Some juveniles are however splitting from the family already in the first winter (after their first autumn migration), and perhaps earlier (AK, unpublished data). In barnacle geese (*Branta leucopsis*), it has been suggested that novel migration strategies arose when families disrupted before the onset of migration [52]. In this species, parental care used to last well into spring migration, but this seems no longer the case since migration was delayed because of changing environmental conditions [53].

### Male-biased switching?

Waterfowl migration systems are also unique in that they show female-biased nest site fidelity [54]. This might be especially pronounced in (partly) capital breeders, like white-fronted geese [55], where it is most important for the female to accumulate reserves by following the green wave in spring [32]. In another study of barnacle geese, the large majority of individuals switching between breeding populations (within the same flyway) were males [17]. In waterfowl, pair formation is generally believed to take place in the wintering grounds or during spring migration, so males tend to follow females to the breeding grounds [54]. However, ring observations in Greenland white-fronted geese (*Anser A. flavirostris*) suggests that pair formation occurs mainly during spring or perhaps even summer [56]. The adult males in which we recorded switching of flyways may have lost their mate and were able to move more freely. The fact that they moved to the population of presently higher reproductive success might be adaptive, but could also have been caused by uneven sample sizes (i.e. more available GPS tracks from the North Sea population).

### Conservation and management implications

Our findings support the notion that the North Sea and Pannonic populations of greater white-fronted geese should be considered as a meta-population complex. Thus, management actions like increased derogation shooting or hunting in the wintering sites [30] of one population can affect the other. Due to its low breeding success, the North Sea population, even if presently stable at high numbers, should be of concern. As has been shown for e.g. Greenland white-fronted geese [13], it might be a cryptic sink that is kept stable by immigration from other wintering sites, like the Pannonic population or others further east.

Furthermore, the high breeding failure in the North Sea population can lead to higher levels of moult migration and, in light of our results, higher flyway exchange rates. Thus, careful monitoring and a quantification of the exchange with the Pannonic population are needed to fully understand the dynamics of this meta-population. This might become more important in the light of presently high levels of climate and habitat change, as population exchange events can increase or decrease in frequency and magnitude [57] due to weather related events [14, 15], Consequently, population dynamics can change, possibly leading to new migration flyways if switching is not complete [14] or complete population shifts if switches are permanent.

## Conclusions

Here we have shown that, at least in waterfowl with their typical wing moult, moult migrations are an important additional aspect when considering migratory connectivity. By doing so, we have indicated where and when previously suggested population exchange might occur [16, 30]. For the two populations of greater white-fronted geese with their differing population development [30], we argue that an understanding of population exchange and migratory connectivity needs to be integrated for management and conservation advice. Specifically the effect of moult migration on population exchange needs to be considered for the two studied goose populations, but also generally for other waterfowl species.

## Abbreviations

g: Gram
GPS: Global positioning system
km: Kilometres
m: Metres
min: Minutes
